# Patient experiences living with pancreatic cancer risk

**DOI:** 10.1186/s13053-015-0034-1

**Published:** 2015-05-21

**Authors:** Meghan Underhill, Donna Berry, Emily Dalton, Jaclyn Schienda, Sapna Syngal

**Affiliations:** 1 Dana-Farber Cancer Institute, Boston, MA USA; 2 Ambry Genetics, (previously Dana-Farber Cancer Institute), Boston, MA USA

**Keywords:** Pancreatic cancer risk, Qualitative research, Patient reported experience

## Abstract

**Background:**

Pancreatic cancer (PancCa) is recognized as a component of many well-described hereditary cancer syndromes. Minimal research has focused on patient needs and experiences living with this risk.

**Purpose:**

To understand the meaning and experience of living with familial PancCa risk and to explore experiences related to screening and prevention of PancCa.

**Methods:**

Participants underwent semi-structured, in-depth interviews. Adults without PancCa and who met familial or hereditary risk criteria were eligible. Thematic analysis was completed on the transcripts in order to identify patterns, consistencies, and differences. Narrative review of existing literature related to women living with hereditary breast and ovarian cancer (HBOC) risk was completed to explore similarities and differences between published findings and our current findings.

**Results:**

Nineteen individuals (9 male, 10 female) participated. Major themes addressed participants’ family experiences with PancCa and PancCa death and the associated grief from the experiences. Family experiences impacted how participants interpreted and approached their own cancer risk and participated in the cancer screening program. Participants wanted to control their cancer risk and sought information and resources to prevent PancCa or PancCa related death. Distress related to risk was not described as constant but occurred around salient time points.

**Conclusion & future implications:**

Study results begin to describe the lived experience of individuals with PancCa risk. Through this research we have uncovered important variables to further understand, measure, and intervene upon in future research. Distress related to risk was not described as ongoing, but occurred around specific and salient time points that brought risk to the forefront. Individuals with familial PancCa risk may have a unique experience compared to other hereditary cancer syndromes due to the high mortality of the disease and uncertainty related to prevention and early detection outcomes.

## Background

In 2030 pancreatic cancer (PancCa) is expected to be the second leading cause of cancer related death in the United States [1]. Of the 46,420 estimated individuals in the U.S. who were diagnosed with PancCa in 2014, 85 % of those diagnosed will die from the disease [2]. If PancCa is stage IA when diagnosed, an individual has a 5-year survival rate of 14 %, compared to 100 % [3] and 92 % [2] in stage I breast and colon cancer, respectively. For those with Stage III and IV PancCa, the 5-year survival rate is only 1–3 % [2]. Because there are few signs and symptoms of PancCa, most patients are diagnosed at a late, incurable stage. While overall rates of cancer and cancer death have been declining, rates of PancCa diagnosis have been increasing 1.5 % per year since 2004 and death rates increased 0.4 % each year from 2004–2008 [4].

Within the general population, an individual has approximately a 1.41 % lifetime risk for developing PancCa [4]. However, PancCa is now considered both a familial risk related cancer and a component of hereditary cancer predisposition syndromes [5]. Based on consensus agreement [6], individuals are considered at high risk for PancCa due to family history if two or more first degree relatives or any three or more relatives, including at least one first degree relative, have a diagnosis of PancCa. Additionally, individuals with Peutz-Jeghers Syndrome and those with *p16*, Hereditary Breast and Ovarian Cancer Syndrome (HBOC) or *BRCA1/2*, *ATM, PALB2,* or Lynch syndrome gene mutations and one or more first degree relatives with PancCa are also considered at risk [4]–[6] due to genetic factors. Individuals who meet these criteria have higher risk burden ranging from a lifetime risk of 3.6 to 40 % [5], [6]. Consequently individuals with elevated risk live with the awareness that if diagnosed with PancCa most will die.

If a person is identified as being at risk for PancCa, a healthy lifestyle, including tobacco cessation [6] or weight loss [4], is recommended. Additionally, consensus based guidelines from the International Cancer of the Pancreas Screening Consortium (CAPS) [6] recommend PancCa screening with endoscopic ultrasound (EUS) or magnetic resonance imaging (MRI). If a lesion that is a precursor to cancer is identified, individuals are recommended to seek care at a specialty care center that has experience with high risk PancCa [6].

With well-established and well-studied cancer syndromes, such as HBOC and hereditary colon cancer syndromes, there is evidence of psychosocial distress associated with cancer risk [7]. Factors such as psychosocial distress, perceptions of cancer risk and cancer worry, and perceived ability to control cancer risk all influence psychosocial outcomes in people with hereditary breast or colon cancers [7]–[15]. Additionally, within these cancer risk syndromes, risk perception is known to impact both health behaviors and quality of life [16].

In contrast, there are limited data regarding patient reported psychosocial outcomes in PancCa. Most studies focus on measuring outcomes at set time points after genetic counseling or surveillance [17]–[19]. These studies have not documented clinically significant distress over time in relation to genetic testing or surveillance. However, those with high risk perception and baseline psychosocial distress did experience distress post-testing and during surveillance. Ethnographic work with 13 individuals with a family history of PancCa or a *BRCA2* mutation demonstrated that limited knowledge about pancreatic risk, insurance coverage, learning of a cancer diagnosis, and the complexity of recommendations were all important components of how individuals approached PancCa screening [18]. Screening is only one component of living with PancCa risk. The magnitude of the potential psychosocial impact on surviving family members is vast and largely unexplored within this domain. Clinicians will not have the tools to assess and then subsequently intervene to improve psychosocial outcomes for individuals and families living with PancCa risk until the context of this experience is described and measured.

Therefore, the purpose of this study was to explore how individuals with high risk for inherited or familial PancCa experience live with this risk. Aims were to: (1) understand what it means to live with the awareness of high risk for inherited or familial PancCa and (2) explore experiences as part of a comprehensive cancer prevention program, including undergoing surveillance procedures for pancreatic cancer.

## Methods

### Sample and recruitment

The Dana-Farber Cancer Institute (DFCI) Institutional Review Board reviewed and approved this study. Participants were recruited from the Gastrointestinal Cancer Genetics and Prevention Program either in person at a scheduled clinic visit or over the telephone by clinical staff that were part of the research team. Eligible participants were over the age of 21 and did not have a PancCa diagnosis. Participants all had personal, genetic, or familial factors that implicated elevated PancCa risk, as verified by medical record review. Specifically, all participants came from a unique family in which two or more members had a diagnosis of PancCa, and were first degree relatives of one of the affected cases, or the participant had a known hereditary cancer syndrome (based on germline genetic testing demonstrating a pathogenic mutation) that is associated with elevated pancreatic cancer risk plus any family history of PancCa. Written informed consent was completed prior to each interview.

### Study design and process

This was a descriptive phenomenological study utilizing qualitative interviews, which aimed for in-depth exploration of an unexplored phenomenon [20]. Data were collected through a single one-on-one, in-person interview. The first author, trained in qualitative methods, completed all interviews. The semi-structured interviews began by asking participants to “Think back to when you first learned that you were at increased risk for PancCa and describe that experience.” The interviewer prompted discussion of the following topics if the participant did not discuss the topic independently: 1) What does PancCa risk mean to you?; 2) What information is important for you to know about PancCa risk?; 3) How do you feel about PancCa screening and what, if any, screenings have you had?; and 4) What advice would you give someone else living with PancCa risk? All participants were asked to discuss any additional relevant information not covered in the questions at the conclusion of the interview. A brief demographic survey was reviewed with each participant and family history data was extracted from the medical record. All interviews were audio recorded and transcribed with participant identifiers removed prior to analysis.

### Analysis

Data analysis was completed in Nvivo 9. Thematic content analysis was used to identify key themes within the interviews [21], [22]. The research team engaged in analysis led by the primary author (M.U.). First, a random subset of the interviews was analyzed independently by the research team and overall themes were identified to develop a coding schema. Then, the primary author reviewed all of the interviews to code for the identified themes. Any additional themes or discrepancies identified were reviewed until consensus was reached and data was re-analyzed. New participants were enrolled and data collected until saturation of themes, or redundancy in data, occurred [22]. After data were analyzed, results were verified through comparison to existing literature and by consensus of the research team which consisted of clinicians and researchers. Measures of central tendency were used to describe the demographic data.

## Results

### Description of sample

Forty eligible patients were approached to participate in the study, sixteen were lost to follow-up, and five declined. Nineteen participants (9 male and 10 female) consented to the study and were interviewed, with interviews lasting 60 to 120 min. Table 1 describes the study sample, including information pertaining to personal, genetic, and family history. As indicated in the table, most participants had familial PancCa risk. The sample had a median of 3 (range 2–6) affected individuals within the family. Cancers of the breast, ovaries, colon, prostate, stomach, and melanoma also were mentioned as occurring within the families; breast and ovarian were most common.

**Table 1:** Description of study sample

		Median (range)
Age (years)		59 (37–80)
		n (%)
Highest level of education	High school	2 (10.5)
	College or some college	5 (26.3)
	Graduate school	12 (63.2)
Familial Cancer History	Hereditary cancer syndrome	6 (31.6)
	Familial pancreatic cancer syndrome	13 (68.4)
Prior medical history	Breast cancer	2 (10.5)
	Endometrial cancer	1 (5.3)
	Chronic pancreatitis	1 (5.3)

### Overview

Three primary themes and nine subthemes were identified in the analysis. Exemplar quotations for each theme are listed in Table 2 and in the results below.

**Table 2:** Themes and exemplar quotations

Theme	Subtheme	Exemplar Quotation	Participant characteristic
Experiencing cancer risk in the family			
	Grieving	“I can never forget this–it’s blazed in my mind. My husband was standing—I had come in the house, walked by him; we were chatting, and he said, “I have something serious to tell you. Your cousin passed away.” Right behind him was my son, who was young. And I just lost it, started crying. I think knowing that it hit our generation, the third generation, it’s very upsetting (crying). …”	60 year old female, familial pancreatic cancer
	Considering own mortality	“…my brother and I kind of half-joke about this a lot, we’re not going to see seventy-five, you know, so this sense of mortality is very present lately. I’ve started to almost countdown backwards…I have thirty years, which is different than I’ve lived, so now I’m going to die quicker than I’ve lived…over-the-hill has passed already…”	40 year old male, *BRCA2* gene mutation, father passed away at age 70 from pancreatic cancer
	Experiencing fear	“My male cousin, first cousin got pancreatic cancer and died when he was fifty three and my aunt…also died at about 78…so it’s just always in the back of my mind… You know, Are we going to die from this? Is this our death sentence waiting for us?—it’s made me very focused on being afraid of cancer in general.”	64 year old female, familial pancreatic cancer
		“I don’t worry about hypertension. I don’t worry about car crashes. I don’t worry about strokes. There’s a false positive that gets created, which is, you know, by being twelve, or fourteen percent likely to die of pancreatic cancer, you get it in your head that you are going to die of pancreatic cancer, like when you’re driving and you focus on a tree, you could go hit it. But the inverse, of course, that there is an eight-six percent chance that you are going to die from something else. And for some reason that isn’t on my mind, and I don’t know why, other than it’s unknown…”	40 year old male, *BRCA2* gene mutation
Seeking a way to control cancer risk			
	Wanting to be taken care of	“I’m not concerned because I have total faith in the doctors. If the cancer shows up they’ll take care of me…”	66 year old female familial pancreatic cancer
	Approaching health	“I try to live healthy, you know? I don’t drink. I don’t smoke.”	62 year old female, pancreatitis
		“So you really do have to live for the day and enjoy it.”	62 year old female, BRCA2 gene mutation, history of breast cancer
		I started exercising—I’m up to once a week—I have lived a sedentary lifestyle… I need to start exercising; I’ve cut back on cigar smoking excessive alcohol. There is a consciousness that if I am only going to make it into my seventies, I want to try to at least be healthy…I don’t want to be decrepit in my sixties if I have only so many years to go. I don’t ever think any of those things are going to help me extend.	40 year old male, *BRCA2* gene mutation, father passed away at age 70 from pancreatic cancer
	Avoiding a similar familial cancer experience	“… it’s not like a heart attack. You don’t die overnight or you don’t die kind of instantly… it’s a real rollercoaster. And you see them slowly losing weight, and losing weight, and losing weight and just sort of becoming you know so terribly frail and thin, you know really to the point where you know they really are skin and bones.	59 year old female, Lynch syndrome
		…I don’t know if it’s somewhat subconscious or whatever that I just seem to feel most comfortable at an overweight weight. I’m trying to pack it on so that I have more to give up as opposed to being the ideal weight, and then getting sick and then losing precious body mass, or organ mass or whatever that at least if I’ve got some extra fat on me then I can burn that off while either I’m trying to get better, or they find a cure or whatever, ifs I can come up with in my brain.”	
		“A friend of mine asked me if you have pancreatic cancer what will you do? And I was like right now I’d say that…I’d get a house on the water, and I’ll read and I’ll just enjoy what I got left, watch the sunsets and watch the sun rises. [the friend asked] You wouldn’t do chemo? You won’t do radiation and all? The way I feel right now, no, because I watched too many people go through all of that.	58 year old male, *BRCA2* gene mutation
Undergoing PancCa screening			
	Doing something to try and catch cancer early	“…if there was ever anything that it (pancreatic cancer screening) would be able to—to be caught early. So that’s—that’s the only—that’s the only hope, right, you can have.”	37 year old female, *BRCA2* gene mutation
	Uncertain benefit of screening	“I’m comforted by the screening, but what’s the end result of the screening? Am I going to lose my pancreas if something is found? That’s the first thing I said to my brother, well, my attitude was I’m not getting screened if they can’t cure it because I don’t want to know that I have it or that I’m going to have it in ten years or highly likely to if there’s no way to cure.”	54 year old male, familial pancreatic cancer; undergoing screening
	Anticipating results	“So around the time of the procedure I start to get a little anxious, like what are we going to learn this year, but once it’s (screening) over …it’s over. “	58 year old female, familial pancreatic cancer

#### Theme 1: experiencing cancer risk in the family

##### Grieving

The initial interview question asked participants to describe the experience of learning about PancCa risk. Most participants responded with a conversation about family experience, while two individuals related a personal cancer (non-PancCa) experience. Even for participants with known genetic mutations predisposing them to cancer, it was the family experience with cancer and death that was paramount to how the participants discussed PancCa risk. Participants were emotional when remembering the loss of their loved ones. Specifically, participants who had close relationships with loved ones that had died of PancCa were grieving the loss. For example, one woman described how when she learned of her own risk for PancCa, she re-evaluated her happiness in her life, work, and living situation. She stated:

[I am]…just kind of making plans for sort of easing out of what’s a very stressful career and doing something completely different, sort of what my heart wants to do instead of what needs to be done to pay the mortgage. I think some of that is driven by sort of…I could have thirty years [or] I could have two days because I could get hit by a car in the street. Pancreatic cancer though, the possibility of that, I think made me stop and think more carefully about it.

Of the 15 participants who described the time frame in which they witnessed PancCa in the family, only two had experiences within the past year. All others told detailed stories of lost loved ones from years, even decades, prior. The four individuals who did not describe a timeframe for witnessing PancCa in the family shared stories in the past tense, not the present.

##### Considering and planning for one’s own mortality

Witnessing multiple family members die of cancer raised the participant’s awareness of the potential for developing cancer or experiencing cancer related death. One participant referred to PancCa as a “death sentence,” which was a common sentiment as no participants had witnessed a loved one survive a PancCa diagnosis. During the interviews, those participants who experienced the death of a loved one with PancCa in the past discussed end of life care wishes and a desire to avoid a similar death experience. One man, with a *BRCA2* gene mutation and a father who had died of PancCa, discussed in detail his end of life care wishes and how they were impacted by the fear of dying the way his father died.

Individuals expressed that it was difficult to discuss mortality and dying of PancCa with their own family and children. Participants had to “compartmentalize” their risk or worry to protect family and planned to avoid having their children experience cancer death as they had in the past.

Pending mortality was discussed within the context of age. Although participants were aware of risks related to heart disease or other chronic illness, due to the significant family experience related to PancCa, participants focused on cancer alone as the cause of future death.

##### Experiencing fear

Participants also discussed fear and worry about developing PancCa. Furthermore, participants feared dying in pain or discomfort, as many had watched loved ones die in that manner. Participants feared placing a burden on loved ones to care for them at the end of life.

This fear and worry was not reported as constant, but experienced at specific and salient time points when the participant was reminded of family experiences or personal risks. These time points included screening or doctors’ visits, anniversaries of loved ones’ deaths, reaching the age of a loved one who died of PancCa, and witnessing another person with cancer, either a friend or a media figure.

#### Theme 2: seeking a way to control cancer risk

##### Wanting to be taken care of

Participants felt “taken care of” by PancCa expert clinicians and “grateful” for the opportunity to be cared for in a way that deceased family members had not experienced. Participants aimed to avoid similar experiences of a PancCa related death, and felt that access to specialists offered more “control” for both early detection and prevention. Participants discussed that having access to specialists was not a resource available to their family members in the past and therefore they were grateful for the resources.

Receiving information for self-care was one aspect of being cared for by clinical experts. Participants sought recommendations related to lifestyle behaviors that were, or were not, important to reduce PancCa risk, and these helped guide self-care decision making. Most participants were also a part of a medical research study and wanted research results shared with them. For some participants finding helpful specific information was challenging because PancCa was not considered a “popular” disease and therefore not the focus of mainstream media. As one participant stated, “…it’s [PancCa] not breast cancer. It’s not…heart disease. It’s not sexy.”

##### Approaching health

Being aware of one’s own PancCa risk led some individuals to live healthfully. Participants discussed diet, exercise, reduced alcohol consumption, and tobacco use as important factors to reduce cancer risk. However, for some the experience of PancCa-related death in the family led to a feeling that life should be lived in the moment and to the fullest. One participant described alcohol consumption as a potential risk factor for PancCa, but reported enjoying alcohol and stated “…it’s not as if I put down the wine.” Living each day to the fullest was mentioned more frequently by those who have had illness in the past, including another cancer diagnosis. Those individuals who had not had a personal diagnosis of cancer spoke more often about modifying risk factors to avoid cancer.

##### Avoiding a similar familial cancer experience

Participants described a need to take action related to their knowledge of PancCa risk, which is what led them to seek care at the cancer center. The goal discussed by participants was to avoid a cancer death experience as seen within the family. There were no stories of survivorship within the participants’ families and all had witnessed one or more loved ones die of the disease. PancCa had meant pain and suffering.

One participant felt so strongly that seeking specialty care was important, that despite a limited budget coming to the cancer center was a priority. Taking part in research was another strategy that participants described to avoid a similar experience and helping other family members avoid the same cancer death. As one woman described “…this [research participation] isn’t really for me. It is for my children and my grandchild.”

#### Theme 3: undergoing PancCa screening

##### Doing something to try and catch cancer early

Engaging in PancCa screening was one way most participants aimed to “do something”. For these participants, screening consisted of EUS and MRI. Participants spoke of “not looking forward to” PancCa screening, but felt it to be a necessary part of their health care. Engaging in screening was a mechanism to be monitored closely and frequently by a clinical expert. PancCa screening was labeled as “prevention” and participants discussed that they hoped to avoid what been their family member's experience and to catch cancer when it was potentially curable.

##### Uncertain benefit of screening

Participants discussed concerns about the efficacy of screening, though most still chose to undergo screening as a mechanism to “be watched.” Only one male participant chose not to undergo cancer screening, stating “… you guys don’t have any real screening device for this yet…” The participant felt that undergoing screening created exposure to risks associated with the procedures and tests and provided little benefit. In contrast, a female participant had undergone a surgical procedure related to findings from PancCa screening and felt that this was an important strategy to reduce PancCa risk.

The outcome of screening over time was also a discussion point within the interviews. One participant wanted to undergo screening, but was not interested unless it led to an ability to cure any identified cancer, and asked “what is the end result?” While this participant chose to undergo screening at the time of the interview, he questioned the uncertainty of what would be done with the information from the screening outcome.

### Anticipating results

Participants discussed experiencing worry or fear while awaiting screening results that may have indicated an abnormal finding. These concerns began in anticipation of the screening appointment. One participant noted irritability or anxiety the week before the screening appointment, yet was unaware of these emotions until it was acknowledged by a coworker. Another recognized that entering the cancer center to complete the screening caused “flashbacks” to familial experiences and fear of finding cancer in themselves at this screening visit. Learning the results of the procedure in a timely manner was helpful to alleviate fear or worry after completing screening.

## Discussion

This study was a descriptive phenomenological study that aimed to begin to describe the meaning and lived experience of living with PancCa risk (aim 1) in the context of a high risk cancer center and screening program (aim 2). Our findings indicate that family experience, specifically an experience with cancer death, was an important component of how the participant approached cancer risk. Participants sought a way to control cancer risk and avoid an experience similar to that of family members through healthy behavior and, for most, screening, despite having fear of the results and being uncertain about the efficacy of the procedure. There was a sense of contradiction and uncertainty related to perceptions of the efficacy of screening and a desire to avoid death from PancCa. Access to specialists gave participants a mechanism to feel “taken care of” by experts was a source of comfort. Participants felt it was challenging to find and interpret information related to preventing and detecting PancCa risk and wanted access to information from health care experts.

Participants in this study felt that living with risk for PancCa was at times an uncertain experience, specifically related to knowing how to prevent or detect PancCa. The familial experience was the most important component of how these participants viewed their own PancCa risk. Stories of long-term survivorship in PancCa did not exist within participants’ families because all of the family members with PancCa died. This contributed to feelings of worry and uncertainty related to PancCa risk, prevention, and screening. In contrast, previous work completed with women at high risk for HBOC and undergoing breast cancer screening and prevention found that women were confident in their ability to detect, treat, and cure breast cancer if found [15]. Therefore, we see the importance of strong clinical recommendations in impacting participant confidence in their ability to live with risk.

Participants talked about not only avoiding cancer, but, more specifically, avoiding a similar cancer experience to that which they had witnessed in the family. Avoidance of cancer death was an important point of discussion and reliving this experience, often involving more than one family member, was emotional to discuss. Similarly, Petrin et al. presented stories from participants who are family caregivers of persons with pancreatic cancer and described how these caregivers experienced worry and fear about their own future cancer risk and wanted to avoid the same cancer related death experience. Participants also discussed a long term psychological impact from caring for a loved one with PancCa [23], which is similar to that described by our participants. Our study provides further evidence that when caring for those with hereditary or familial cancer risk, care needs to extend beyond just identifying and managing risk, but to acknowledgement of the grief associated with losing loved ones to cancer.

Within this sample, participants described a psychosocial response to living with familial pancreatic cancer risk, such as worry and fear, which has been reported in previous literature [24]. These psychological responses were not constant, occurred over the course of the individual’s life, and were present during times that caused the person to remember familial experiences with PancCa. This brings to light the need for psychosocial support for persons living with this risk, not only for managing their own risk, but in coming to terms with a significant familial cancer experience. A similar need has been reported previously and, in a study of 248 Dutch participants with HBOC or HBOC risk, it was reported that individuals may need psychological support regardless of having a “diagnosed” psychological disorder [25]. Only one third of individuals in the Dutch study who requested psychosocial support received support, which provides further evidence of a potential gap in care for those with hereditary cancer risk that, as we have identified, includes those with familial pancreatic cancer risk.

Previous literature supports that at risk individuals are willing to take part in pancreatic cancer risk reduction and screening interventions [24], [26]. Our findings present background as to why participants may be receptive to these interventions and demonstrate the salience of family experience on influencing health choices.

Participants sought ways to control cancer risk and sought care and support from clinical experts. For this study, care was sought from the comprehensive cancer prevention program. Participants described feeling grateful for having access to this resource and also reported that they felt that they had fewer resources compared to people with other cancer risk. The screening and prevention mechanisms, and what to do with the resultant information, for PancCa are evolving, and participants shared uncertainty related to outcomes associated with both lifestyle choices and screening EUS or MRI. In light of the lack of evidence which fully supports screening, participants relied on clinical experts to guide them in making health choices. It is important that clinical research continues to test these mechanisms for screening so that clinicians can have the tools to help fully inform their patients.

### Implications

In this study we began to understand the lived experience of persons with high risk for PancCa. From this initial study, we now understand important factors that will be necessary components of future practice and research endeavors. Practice related implications include a need to provide information to and support access for individuals living with high risk for PancCa. This includes not only genetic risk related information, but also information about self-care benefits, screening, and available psychosocial support services to deal with grief associated with familial loss.

Due to our findings that participants in this study had cancer related fear and worry that were intermittent and would flare up at certain times, individuals with high PancCa risk may benefit from supportive psychosocial intervention targeted to specific times, such as the weeks prior to a screening appointment. Additionally, this fear and worry often stemmed from witnessing family members die of pancreatic cancer. Therefore, assessing if an individual had a family death experience related to PancCa could help identify psychosocial needs and will be an important variable to measure in future research.

Implications also include ensuring psychosocial needs are being adequately measured in future research and that investigators work to identify patient-centered time-points to capture this information. The ultimate goal of this work would be to develop a supportive care intervention tailored to the needs of the participants. A crucial component of tailoring would be to confirm that the intervention is being administered at a time that is impactful to the participant.

These individuals living with familial PanCa risk were highly engaged in the health care system, and actively seeking self-care information. Lawson & Flocke describe this as a “teachable moment” where individuals experience a significant life event, such as witnessing a family member die of cancer or learning of one’s own risk for cancer, and are more likely to make a personal change [27]. Breitkopf and colleagues further validate this model in a sample of family members of persons with colo-rectal cancer and find that participants who are family members of individuals who have or had a diagnosis of colorectal cancer are more willing to take part in a cancer prevention intervention [28]. Therefore, future research interventions targeting the population of caregivers of individuals with PancCa could have the potential to positively impact both medical and psychosocial outcomes. Future research should also aim to include those who do not choose screening to learn key differences in experiences between those who do and do not choose screening and also those from a more diverse background.

### Limitations

Our data are limited to stories from individuals who agreed to participate and therefore might be different from others who did not volunteer. Additionally, all of the participants were receiving care at an academic cancer center while findings may be different at other sites. We acknowledge that the experiences of individuals taking part in a high risk cancer prevention program may be different from experiences in other clinical settings. Most work with this population is currently being done in academic medical centers and therefore this study is just a first step in describing the needs of this population. Future work will need to expand recruitment to outside of the academic setting. Finally, the participants in this study were well educated with limited cultural or racial variability and therefore findings may not reflect the reports of a more diverse sample.

## Conclusion

Study results begin to describe the lived experience of individuals with PancCa risk. Through this research we have uncovered important variables to further understand, measure, and intervene upon in future research, such as fear, worry, and family experience. Distress related to risk was not described as ongoing, but occurred around specific and salient time points that brought risk to the forefront. Individuals with familial PancCa risk may have a unique experience compared to other hereditary cancer syndromes due to the high mortality of the disease and uncertainty related to prevention and early detection outcomes.
